# Peer Review of “Financial Feasibility of Developing Sustained-Release Incrementally Modified Drugs in Thailand’s Pharmaceutical Industry: Mixed Methods Study”

**DOI:** 10.2196/78090

**Published:** 2025-07-01

**Authors:** Parnnaphat Luksameesate

**Affiliations:** 1 Chulalongkorn University, 254 Phaya Thai Rd, Bangkok, Thailand, 66 2 218 2000

**Keywords:** financial, economics, R&D, research and development, surveys, interviews, costs, revenue, policies, drugs, pharmaceuticals

*This is the peer-review report for “Financial Feasibility of Developing Sustained-Release Incrementally Modified Drugs in Thailand’s Pharmaceutical Industry: Mixed Methods Feasibility Study.”*

## Round 1 Review

### General Comments

This paper [1] provides valuable insights into how the Thai pharmaceutical industry should prepare for future developments. The results can be used as a reference to support decision-making and to guide the definition of regulations and processes in Thailand.

### Specific Comments

#### Major Comments

1. Methods: Could you elaborate on how the 5 incrementally modified drug (IMD) experts were selected? Additionally, why was the number of experts limited to 5?

2. Tables 1 and 2: Please replace the term “Literature Review” with the specific author names and the corresponding year (Anno Domini).

3. Table 3: The values of US $1.46 million and US $18.6 million refer to the research and development costs only, correct? These values do not reflect the total cost of developing IMDs (refer to Table 2).

4. Since most of the numbers come from expert input, how do you ensure that these numbers are valid and accurately reflect real-world situations? It may be helpful to provide more information about the characteristics and qualifications of the key informants to support their credibility.

#### Minor Comments

5. Please ensure that all abbreviations are defined the first time they appear in the document. For example, “IMD” should be written out as “Innovative Medical Devices (IMD)” when it is first mentioned, particularly in the introduction.

## Abbreviations

IMD: incrementally modified drug
